# Allowing Physicians to Choose the Value of Compensation for Participation in a Web-Based Survey: Randomized Controlled Trial

**DOI:** 10.2196/jmir.3898

**Published:** 2015-07-29

**Authors:** Alison E Turnbull, Cristi L O'Connor, Bryan Lau, Scott D Halpern, Dale M Needham

**Affiliations:** ^1^ School of Medicine Division of Pulmonary and Critical Care Medicine Johns Hopkins University Baltimore, MD United States; ^2^ Bloomberg School of Public Health Department of Epidemiology Johns Hopkins University Baltimore, MD United States; ^3^ Outcomes After Critical Illness and Surgery Group Johns Hopkins University Baltimore, MD United States; ^4^ Perelman School of Medicine Division of Pulmonary, Allergy and Critical Care University of Pennsylvania Philadelphia, PA United States; ^5^ Perelman School of Medicine Department of Biostatistics and Epidemiology University of Pennsylvania Philadelphia, PA United States; ^6^ School of Medicine Department of Physical Medicine and Rehabilitation Johns Hopkins University Baltimore, MD United States

**Keywords:** data collection, monetary incentives, cash, physicians, electronic questionnaire, survey design, response rate

## Abstract

**Background:**

Survey response rates among physicians are declining, and determining an appropriate level of compensation to motivate participation poses a major challenge.

**Objective:**

To estimate the effect of permitting intensive care physicians to select their preferred level of compensation for completing a short Web-based survey on physician (1) response rate, (2) survey completion rate, (3) time to response, and (4) time spent completing the survey.

**Methods:**

A total of 1850 US intensivists from an existing database were randomized to receive a survey invitation email with or without an Amazon.com incentive available to the first 100 respondents. The incentive could be instantly redeemed for an amount chosen by the respondent, up to a maximum of US $50.

**Results:**

The overall response rate was 35.90% (630/1755). Among the 35.4% (111/314) of eligible participants choosing the incentive, 80.2% (89/111) selected the maximum value. Among intensivists offered an incentive, the response was 6.0% higher (95% CI 1.5-10.5, *P*=.01), survey completion was marginally greater (807/859, 94.0% vs 892/991, 90.0%; *P*=.06), and the median number of days to survey response was shorter (0.8, interquartile range [IQR] 0.2-14.4 vs 6.6, IQR 0.3-22.3; *P*=.001), with no difference in time spent completing the survey.

**Conclusions:**

Permitting intensive care physicians to determine compensation level for completing a short Web-based survey modestly increased response rate and substantially decreased response time without decreasing the time spent on survey completion.

## Introduction

Understanding the opinions and practices of health care providers is essential for clinical research [1]. However, surveys of health care providers are plagued by declining response rates [2-4], and techniques for increasing response rates in the general public [5] frequently fail to motivate physicians [6]. Although low response rates do not necessarily bias results [7,8], they do increase the potential for nonresponse bias, and hamper publication [2,9-11].

While physician response rates are declining, inexpensive tools for conducting sophisticated Web-based electronic surveys are flourishing. Although physician response rates to electronic surveys have generally been lower than to postal surveys, many trials comparing postal versus electronic surveys were conducted a decade ago and targeted community-based physicians in regions where high-speed Internet access was unreliable or nonexistent [12-15]. As high-speed Internet access becomes ubiquitous and health care providers become more comfortable with Web-based technologies, electronic surveys have the potential to provide researchers with unique tools, including instant compensation for participation and data about how physicians interact with surveys.

A major challenge when designing a survey is determining the appropriate level of financial compensation required to incentivize participation [16-18]. Allowing respondents to choose how much they wish to be compensated provides insight into participant motivation and may maximize the cost-effectiveness of incentives by not spending funds on participants who do not require a large incentive to respond. An additional challenge posed by electronic surveys is the inability to provide prepayment. Prepayment in postal surveys, traditionally achieved by including cash in the survey envelope, is associated with significantly greater response rates among surveys of physicians versus providing payment contingent on survey completion [16,19,20].

We combined three techniques to address these challenges. First, we invited physicians to select their preferred level of instant compensation, up to US $50, for completing a short, electronic survey. Second, we attempted to engender altruism by reminding physicians that the study was funded by a limited student budget. Finally, compensation was only promised to the first 100 respondents, making it a scarce, time-limited incentive. To assess whether these three combined techniques affected response rate, time to response, survey completion rate, and time spent completing the electronic survey, we designed a randomized controlled trial of respondent-selected compensation.

## Methods

### Study Design

A previously described database of academic intensivists was used to recruit faculty from US hospitals with training programs accredited by the Accreditation Council for Graduate Medical Education in Internal Medicine-Critical Care Medicine, Anesthesiology-Critical Care Medicine, and Surgical Critical Care [21]. The database was updated in 2012 to include demographic and electronic contact information for 2482 physicians. Physicians were excluded from randomization if they (1) lacked electronic contact information (285/2482, 11.48%), (2) had been invited to participate in a pilot study (268/2482, 10.80%), (3) had made a previous request not to be contacted (76/2482, 3.06%), or (4) contributed to study design or survey development (3/2482, 0.12%). The remaining 1850 intensivists were potentially eligible to participate in a randomized trial of an intervention to increase communication about life support with families of critically ill patients [22], administered using the Qualtrics Web-based survey platform [23]. The Institutional Review Board (IRB) of Johns Hopkins School of Medicine approved the study. Intensivists were notified that survey completion served as consent to participate in the trial.

Randomization was blocked on intensivist sex, specialty—medicine, anesthesiology, or surgery—years since completing residency, and geographic region of residency [24,25]. Within each block, 45% of eligible intensivists were randomly assigned to the group with the ability to select their preferred level of compensation as an incentive to participate.

On November 20, 2012, each randomized intensivist was sent an invitation by email containing a unique link to the survey. All invitations included the survey topic, number of questions, expected time required to complete the survey (5 minutes), IRB approval, study confidentiality, number of follow-up/reminder emails for nonresponders, planned date for study closing (December 20, 2012), and names and affiliations of study investigators. Invitations for intensivists randomized to receive an incentive to participate also included the following text:

In appreciation for your participation, the first 100 respondents to complete the survey will be offered an Amazon.com gift code at the end of the survey. The code can be redeemed immediately for any amount up to $50. In selecting the compensation amount, please consider that this is a PhD thesis project being funded by a limited student budget.

Reminder emails were sent to all intensivists who had not completed the survey on days 13, 22, and 28 following the initial invitation. In each of the reminder emails, intensivists randomized to the incentive group were informed that funds for incentives were still available. Because not all respondents chose to take the full amount available, there were sufficient funds to offer the incentive to more than 100 respondents. To establish survey eligibility, participants were first asked if they had treated patients in the intensive care unit (ICU) setting during the previous 2 years. Those who had were then asked one question about practice history followed by 10 screens, each containing a brief clinical scenario for review.

Participants randomized to the incentive intervention who completed the survey had the option of entering the amount they wished to spend at Amazon.com [26] up to US $50 using the Amazon Gift Codes On Demand service, which allows study participants to claim incentives instantly. Among those participants who elected to receive one, incentives were claimed on the Amazon.com website. The value of created incentives was instantly deducted from a study fund containing US $5000. Study investigators could not access information on goods purchased by participants or the timing of purchases made using gift codes.

### Statistical Analysis

The primary outcome measure was the difference in survey response rate between trial arms. Response rates were calculated in accordance with response rate three (RR3), defined by the American Association for Public Opinion Research guidelines for Internet surveys of specifically named persons [27] as follows:

RR3 = I/(I + P + *eC*[UH + UO] + *eI*[UH + UO])

The RR3 is equal to the number of eligible participants who responded to all survey questions (I) divided by the sum of eligible participants who responded to all survey questions (I), eligible participants who answered the eligibility screening question but did not answer all survey questions (P), and the estimated proportion in the control arm (*eC)* and intervention arm (*eI)* of nonresponders (UH) and responders with unknown eligibility (UO) who were eligible. The proportions of eligible nonresponders and responders with unknown eligibility were estimated based on the proportion of responders in each arm of the trial who answered the screening question and were known to be eligible.

Secondary outcome measures were defined as follows: survey completion rate (ie, number of intensivists known to be eligible who answered all survey questions divided by the number of eligible intensivists who clicked the link to the Web-based survey), time to response (ie, time of survey completion minus the time the initial email was sent among intensivists who completed the survey), and time spent completing survey (ie, time of survey completion minus time that the link in the invitation email was clicked by eligible intensivists).

Analyses were performed using the R programming language version 3.0.1 (Vienna, Austria) [28] using two-sided significance tests, with *P*<.05 indicating statistical significance, and data were analyzed on an intention-to-treat basis. Hypothesis tests for differences in proportion were performed using Pearson’s chi-square test. Fisher’s exact test was used when a cell within a contingency table contained fewer than 10 observations. Confidence intervals for differences in proportions were calculated using the Wald interval. Differences in the distribution of continuous variables were assessed using the Wilcoxon-Mann-Whitney test [29].

## Results

### Overview

**Figure 1:**
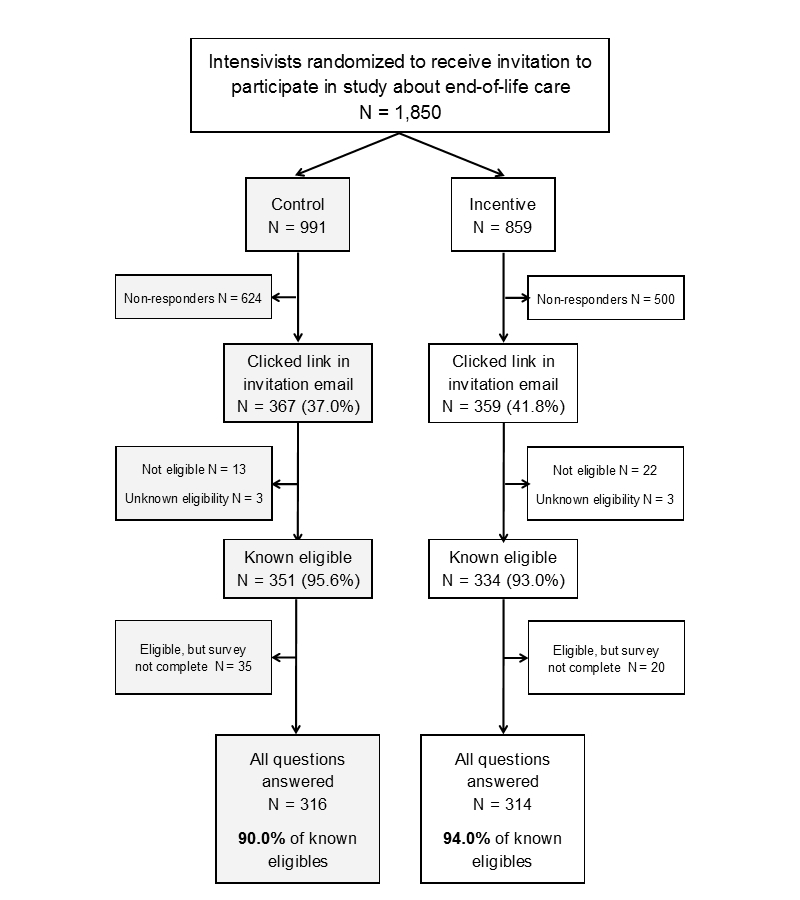
Study profile. Eligible participants had treated patients in the intensive care unit (ICU) setting during the last 2 years.

The overall response rate was 35.90% (630/1755), with 92.0% (630/685) of eligible respondents answering all questions in the survey (see Figure 1 and Table 1). A total of 13 out of 991 (1.3%) respondents in the control arm and 22 out of 859 (2.6%) respondents in the incentive arm indicated that they had not treated patients in the ICU setting in the last 2 years and were deemed ineligible. Among the 55 known eligible respondents who did not complete the survey, 32 (58%) answered the screening question and the question about practice history, but did not answer any of the questions related to the brief clinical scenarios. The remaining 23 (42%) participants responded to a median of 4 scenarios (interquartile range [IQR] 3-6). The median time to response was 3.4 days (IQR 0.3-22.0) and the median amount of time eligible intensivists spent completing the survey was 3.9 minutes (IQR 2.5-5.5). Among eligible intensivists invited to participate, 80.49% (1489/1850) were male, 63.30% (1171/1850) specialized in internal medicine, and the median number of years since completing initial residency was 20 (IQR 13-28). The characteristics of intensivists randomized to the control versus incentive groups were similar (see Table 2).

Based on the RR3 equation, the overall response rate was equal to 35.90%:

(316+314)/([316+314]+[35+20]+ .96 [624+3]+.93[500+3])= 630/1755 = 35.90%

The control arm response rate was equal to 33.2%:

316/(316+35+ .96 [624+3])= 316/953=33.2%

The incentive arm response rate was equal to 39.2%:

314/(314+20+ .93[500+3])= 314/802=39.2%

**Table 1:** Response rate calculation values using response rate three (RR3)^a^.

Term	Definition	Control arm (n=991), n (%) or n/n (proportion)	Incentive arm (n=859), n (%) or n/n (proportion)
I	Eligible participants who answered all survey questions, n (%)	316 (31.9)	314 (36.6)
P	Participants who answered the eligibility question but did not answer all survey questions, n (%)	35 (3.5)	20 (2.3)
UH	Nonresponders, n (%)	624 (63.0)	500 (58.2)
UO	Responders with unknown eligibility, n (%)	3 (0.3)	3 (0.3)
*eC*	Estimated proportion of eligible participants in the control arm, n/n (proportion)	351/367 (0.96)	N/A^b^
*eI*	Estimated proportion of eligible participants in the incentive arm, n/n (proportion)	N/A	334/359 (0.93)

^a^RR3 = I/(I + P + *eC*[UH + UO] + *eI*[UH + UO]). RR3 is defined by the American Association for Public Opinion Research guidelines for Internet surveys of specifically named persons [27].

^b^Not applicable (N/A).

**Table 2:** Characteristics of the study population^a^.

Variable		Control (n=991), n (%) or median (IQR^b^)	Incentive (n=859), n (%) or median (IQR)
Male, n (%)		786 (79.3)	703 (81.8)
**Specialty, n (%)**			
	Medicine	615 (62.1)	556 (64.7)
	Surgery	204 (20.6)	170 (19.8)
	Anesthesia	172 (17.4)	133 (15.5)
Years since residency, median (IQR)		20 (13-28)	20 (13-27)
Years since residency not reported, n (%)		156 (15.7)	134 (15.6)
**Region of residency** ^c^ **, n (%)**			
	Northeast	314 (32.0)	291 (33.9)
	Midwest	215 (21.7)	189 (22.0)
	South	218 (22.0)	192 (22.4)
	West	113 (11.4)	97 (11.3)
	International	55 (5.5)	36 (4.2)
	Unknown	73 (7.4)	54 (6.3)

^a^Percentages may not sum to 100% due to rounding.

^b^Interquartile range (IQR).

^c^Region defined according to US census region.

### Primary and Secondary Outcomes

The overall response rate among eligible intensivists offered an incentive was 39.2% (314/802) versus 33.2% (316/953) in the control group (*P*=.01) (see Figure 1). The proportion of eligible respondents answering all survey questions was modestly greater in the incentive group (807/859, 94.0%) versus the control group (892/991, 90.0%) (*P*=.06). Among the known eligible participants who did not complete the survey, 65% (13/20) in the incentive group and 54% (19/35) in the control group did not answer questions related to the brief clinical scenarios (*P*=.44). In contrast to these relatively small effects on response, the incentive was associated with a large reduction in median time to response among responders (0.8 days for incentive group and 6.6 days for control group, *P*=.001; see Table 3). The median time required to complete the survey was 3.9 minutes in each group (*P*=.56).

**Table 3:** Survey outcomes by intervention arm.

Survey outcomes	Control (n=991), n (%) or median (IQR^a^)	Incentive (n=859), n (%) or median (IQR)	*P* ^b^
Response rate^c^, n (%)	329 (33.2)	337 (39.2)	.01
Eligible responders completing survey, n (%)	892 (90.0)	807 (94.0)	.06
Days to response among eligible responders, median (IQR)	6.6 (0.3-22.3)	0.8 (0.2-14.4)	.001
Minutes spent completing survey, median (IQR)	3.9 (2.5-5.5)	3.9 (2.4-5.5)	.56

^a^Interquartile range (IQR).

^b^Calculated using the chi-square test for proportions and the Wilcoxon-Mann-Whitney test for continuous variables.

^c^Response rate calculated in accordance with response rate three (RR3) defined by the American Association for Public Opinion Research guidelines for Internet surveys of specifically named persons [27].

### Incentive Amount

Out of 314 intensivists who answered all survey questions and were offered an incentive, 111 (35.4%) chose to create one (see Table 4). All who chose to accept an incentive were able to do so, as US $95 remained at study end from the US $5000 originally budgeted for incentives. Overall, 80.2% (89/111) of accepted incentives were for the maximum value of US $50. Among the 22 intensivists out of 111 (19.8%) who chose incentives worth less than US $50, the median value was US $20 (IQR $11-$25). Intensivists randomized to the incentive group who accepted, versus did not accept, incentives completed their first residency more recently (median years since residency 15 vs 19, respectively; *P*=.004). A greater proportion of male versus female intensivists chose incentives (99/259, 38.2% versus 12/55, 22%, respectively; *P*=.03).

**Table 4:** Participant characteristics and survey outcomes within the incentive arm of the trial, by participant response to financial incentive^a^.

Variable		Incentive declined (n=203), n (%) or median (IQR^b^)	Incentive claimed (n=111) , n (%) or median (IQR)	*P* ^c^
Male, n (%)		160 (78.8)	99 (89.2)	.03
**Specialty, n (%)**				.17
	Medicine	128 (63.1)	68 (61.3)	
	Surgery	43 (21.2)	17 (15.3)	
	Anesthesia	32 (15.8)	26 (23.4)	
Years since residency, median (IQR)		19 (13-27)	15 (10-22)	.004
**Region of residency** ^d^ **, n (%)**				.63
	Northeast	68 (33.5)	33 (29.7)	
	Midwest	55 (27.1)	24 (21.6)	
	South	44 (21.7)	30 (27.0)	
	West	25 (12.3)	10 (9.0)	
	International	6 (3.0)	4 (3.6)	
	Unknown	5 (2.5)	10 (9.0)	
Days to response among responders, median (IQR)		0.5 (0.2-13.4)	2.9 (0.3-18.5)	.10
Minutes spent completing survey, median (IQR)		3.7 (2.4-5.5)	4.2 (2.6-5.5)	.66

^a^Percentages may not sum to 100% due to rounding.

^b^Interquartile range (IQR).

^c^Calculated using Fisher’s exact test, the chi-square test for proportions, and the Wilcoxon-Mann-Whitney test for continuous variables.

^d^Region defined according to US census region.

## Discussion

### Principal Findings

In a national randomized trial of 1850 academic intensivists, permitting these physicians to choose their preferred level of financial compensation for participating in a short Web-based survey resulted in a 6.0% (95% CI 1.5-10.5, *P*=.01) absolute increase (15.3% relative increase) in response rate, a 3.9% absolute increase in survey completeness, and a faster response time (0.8 vs 6.6 days), with no impact on the time spent completing the survey. Although 66.7% of intensivists offered compensation did not take it, those who did accept it generally took the maximum US $50 amount that was available to them.

Among intensivists offered an incentive, the only respondent characteristics associated with taking it was time since completing residency and gender. More recent graduates of medical training are likely to have lower salaries, higher educational debt levels, and greater electronic expertise, making US $50 more valuable and accessible. Although previous studies have found male health care workers to be less likely to respond to surveys than women [2,10], a sex-based difference in response to compensation has not been commonly reported in prior literature and merits greater investigation. The observed association between instant compensation and time to response is likely to have been influenced by the perceived scarcity of the compensation (ie, the invitation email said the incentive would be offered to the first 100 respondents) and the proximity of the study timing to annual holiday spending in December. This enhanced desirability of scarce resources is a well-known psychological effect [30], and an example of the larger phenomenon of loss aversion [31].

Shortening the time to survey response decreases the number of reminder or follow-up contacts required. Sending fewer reminders saves time and money when surveys are administered by post. Additionally, previous work suggests that late responders often differ from early responders both demographically and in their survey responses [32-35]. Techniques that recruit physicians who intend to respond, but are prone to delaying participation, help ensure their unique perspectives are represented in the study sample.

The fact that relatively few intensivists (33.3%) took the incentive may have meant that most respondents were sufficiently interested in the survey topic not to require any further motivation for participation, or that the survey was short enough—median completion time was 3.8 minutes—that most respondents did not require reimbursement. Participants who took the incentive may have been less interested in the survey topic, but this difference in interest did not lead these participants to spend less time considering or answering survey questions. Efficiently incentivizing participants to thoughtfully answer all questions may be more important for lengthier research questionnaires, although recent trials have reported no association between survey length and physician response for either postal [36-38] or Web-based surveys [39]. Decisions about whether to take the incentive also may have been influenced by altruistic or sympathetic sentiments created by disclosing the limited funding available for this student thesis project.

As physician response rates decline, leveraging available funds to incentivize survey participation becomes increasingly important. Allowing physicians in the incentive group to decide whether they wished to be compensated and explicitly mentioning that only the first 100 respondents could take the incentive allowed us to offer the full US $50 to the 859 targeted intensivists who were potentially motivated by financial gain, despite a budget of only US $5000. There are two alternative incentive strategies to consider. The first is providing US $50 to all 314 eligible respondents who completed the survey. This would have cost US $15,700. Compared to this incentive strategy, our approach produced an absolute savings of US $10,845 and a relative savings of 69.08%. The second incentive strategy is to provide a flat incentive of US $50 to the 111 eligible respondents who requested compensation which would have cost US $5500. By permitting these 111 respondents to determine their preferred compensation level up to a maximum of US $50, the total value of compensation requested was US $4855. Compared to the second strategy, providing the option to choose the value of compensation to those who requested it resulted in an absolute savings of US $645 and a relative savings of 11.73%.

Determining the appropriate amount of compensation to offer for survey completion remains challenging. Given that the vast majority of respondents who elected compensation took the maximum amount suggests that US $50 may not have been viewed as sufficient by the majority of intensivists requiring a financial incentive to participate in this very short survey. Future studies with the ability to offer greater incentives and, thus, subject to less of a ceiling effect could provide insight into the distribution of preferred compensation for survey participation among physicians.

It is important to consider the ethical and practical ramifications of perceived scarcity and providing different levels of financial compensation to members of the same study cohort. If provided as remuneration for a participant’s time, failing to provide sufficient compensation may be ethically untenable or impact data quality. In such cases, a preferable strategy would be to offer an ethically acceptable level of remuneration for all participants completing the survey and to permit the subset of participants who respond most quickly to choose a preferred level of compensation beyond the minimum remuneration level. In such cases, study investigators would need sufficient funds to cover the maximum possible cost of the total compensation and would effectively be incentivizing prompt responses.

### Limitations and Strengths

A potential limitation of our study is the generalizability of our results to other groups of physicians and other health care providers. Additionally, as a bundled intervention comprised of three techniques to optimize survey response, we cannot isolate the impact of any one technique or detect any potential synergistic effects. Study strengths include the use of a national database of academic intensivists containing demographic information on physicians who are almost certain to have regular Internet access, and an electronic survey platform that provided important details regarding survey completion. A study of health care providers offered a gift card to a retail store chain in a postal survey found that provider response decreased in proportion to distance from the nearest store [40]. By offering an incentive that can be used to make Web-based purchases, it is unlikely that the decision to create an incentive was influenced by concerns about proximity to a physical location.

### Conclusions

In conclusion, in this randomized controlled trial of 1850 US academic intensivists, giving physicians a time-limited opportunity to choose how much they wished to be compensated for participation in a brief, Web-based survey was associated with a small increase in response rate and a substantial decrease in time to response, without any decrease in how long physicians spent in completing the survey.

## Appendix

Multimedia Appendix 1 CONSORT-EHEALTH checklist V1.6.2 [41].

## Abbreviations

eC: the estimated proportion in the control arm
eI: the estimated proportion in the intervention arm
I: number of eligible participants who responded to all survey questions
ICU: intensive care unit
IQR: interquartile range
IRB: Institutional Review Board
N/A: not applicable
P: eligible participants who answered the eligibility screening question but did not answer all survey questions
RR3: response rate three
UH: nonresponders
UO: responders with unknown eligibility

## References

[1] Klabunde CN, Willis GB, McLeod CC, Dillman DA, Johnson TP, Greene SM, Brown ML (2012). Improving the quality of surveys of physicians and medical groups: a research agenda. Eval Health Prof.

[2] Cull WL, O'Connor KG, Sharp S, Tang SS (2005). Response rates and response bias for 50 surveys of pediatricians. Health Serv Res.

[3] Cook JV, Dickinson HO, Eccles MP (2009). Response rates in postal surveys of healthcare professionals between 1996 and 2005: an observational study. BMC Health Serv Res.

[4] Cho YI, Johnson TP, Vangeest JB (2013). Enhancing surveys of health care professionals: a meta-analysis of techniques to improve response. Eval Health Prof.

[5] Edwards PJ, Roberts I, Clarke MJ, Diguiseppi C, Wentz R, Kwan I, Cooper R, Felix LM, Pratap S (2009). Methods to increase response to postal and electronic questionnaires. Cochrane Database Syst Rev.

[6] Flanigan TS, McFarlane E, Cook S (2008). Conducting survey research among physicians and other medical professionals: a review of current literature. Proceedings of the American Association for Public Opinion Research 63rd Annual Conference.

[7] Asch DA, Jedrziewski MK, Christakis NA (1997). Response rates to mail surveys published in medical journals. J Clin Epidemiol.

[8] Halpern SD, Asch DA (2003). Commentary: Improving response rates to mailed surveys: what do we learn from randomized controlled trials?. Int J Epidemiol.

[9] Montori VM, Leung TW, Walter SD, Guyatt GH (2005). Procedures that assess inconsistency in meta-analyses can assess the likelihood of response bias in multiwave surveys. J Clin Epidemiol.

[10] Listyowardojo TA, Nap RE, Johnson A (2011). Demographic differences between health care workers who did or did not respond to a safety and organizational culture survey. BMC Res Notes.

[11] Halbesleben JR, Whitman MV (2013). Evaluating survey quality in health services research: a decision framework for assessing nonresponse bias. Health Serv Res.

[12] McMahon SR, Iwamoto M, Massoudi MS, Yusuf HR, Stevenson JM, David F, Chu SY, Pickering LK (2003). Comparison of e-mail, fax, and postal surveys of pediatricians. Pediatrics.

[13] Leece P, Bhandari M, Sprague S, Swiontkowski MF, Schemitsch EH, Tornetta P, Devereaux PJ, Guyatt GH (2004). Internet versus mailed questionnaires: a controlled comparison (2). J Med Internet Res.

[14] Seguin R, Godwin M, MacDonald S, McCall M (2004). E-mail or snail mail? Randomized controlled trial on which works better for surveys. Can Fam Physician.

[15] Nicholls K, Chapman K, Shaw T, Perkins A, Sullivan MM, Crutchfield S, Reed E (2011). Enhancing response rates in physician surveys: the limited utility of electronic options. Health Serv Res.

[16] Asch DA, Christakis NA, Ubel PA (1998). Conducting physician mail surveys on a limited budget. A randomized trial comparing $2 bill versus $5 bill incentives. Med Care.

[17] Halpern SD, Ubel PA, Berlin JA, Asch DA (2002). Randomized trial of 5 dollars versus 10 dollars monetary incentives, envelope size, and candy to increase physician response rates to mailed questionnaires. Med Care.

[18] Keating NL, Zaslavsky AM, Goldstein J, West DW, Ayanian JZ (2008). Randomized trial of $20 versus $50 incentives to increase physician survey response rates. Med Care.

[19] Edwards P, Roberts I, Clarke M, DiGuiseppi C, Pratap S, Wentz R, Kwan I (2002). Increasing response rates to postal questionnaires: systematic review. BMJ.

[20] Leung GM, Johnston JM, Saing H, Tin KY, Wong IO, Ho LM (2004). Prepayment was superior to postpayment cash incentives in a randomized postal survey among physicians. J Clin Epidemiol.

[21] Halpern SD, Hussen SA, Metkus TS, Ward NS, Luce JM, Curtis JR (2007). Development of an e-mail database of US intensive care physicians. J Crit Care.

[22] Turnbull AE, Krall JR, Ruhl AP, Curtis JR, Halpern SD, Lau BM, Needham DM (2014). A scenario-based, randomized trial of patient values and functional prognosis on intensivist intent to discuss withdrawing life support. Crit Care Med.

[23] Qualtrics.

[24] Imai K, King G, Stuart E (2008). Misunderstandings between experimentalists and observationalists about causal inference. J R Stat Soc Ser A Stat Soc.

[25] US Census Bureau.

[26] Amazon.com.

[27] The American Association for Public Opinion Research (2011). Standard Definitions: Final Dispositions of Case Codes and Outcome Rates for Surveys. 7th edition.

[28] The R Core Team (2013). R: A Language and Environment for Statistical Computing.

[29] Hollander M, Wolfe DA (1999). Nonparametric Statistical Methods. 2nd edition.

[30] Lynn M (1991). Scarcity effects on value: a quantitative review of the commodity theory literature. Psychology & Marketing.

[31] Kahneman D, Knetsch J, Thaler R (1991). Anomalies: the endowment effect, loss aversion, and status quo bias. J Econ Perspect.

[32] Voigt LF, Koepsell TD, Daling JR (2003). Characteristics of telephone survey respondents according to willingness to participate. Am J Epidemiol.

[33] Chretien J, Chu LK, Smith TC, Smith B, Ryan MA, Millennium Cohort Study Team (2007). Demographic and occupational predictors of early response to a mailed invitation to enroll in a longitudinal health study. BMC Med Res Methodol.

[34] Prabhakaran J, Spera C, Leach L, Foster R (2010). Differences in early and late responders: findings from a military Web-based community survey. Proceedings of the American Association for Public Opinion Research (AAPOR) 65th Annual Conference.

[35] Leadbetter S, Hawkins NA, Scholl LE, McCarty FA, Rodriguez JL, Freedner-Maguire N, Alford SH, Bellcross CA, Peipins LA (2013). Recruiting women for a study on perceived risk of cancer: influence of survey topic salience and early versus late response. Prev Chronic Dis.

[36] Glidewell L, Thomas R, MacLennan G, Bonetti D, Johnston M, Eccles MP, Edlin R, Pitts NB, Clarkson J, Steen N, Grimshaw JM (2012). Do incentives, reminders or reduced burden improve healthcare professional response rates in postal questionnaires? two randomised controlled trials. BMC Health Serv Res.

[37] Bolt E, van der Heide A, Onwuteaka-Philipsen B (2014). Reducing questionnaire length did not improve physician response rate: a randomized trial. J Clin Epidemiol.

[38] Cottrell E, Roddy E, Rathod T, Thomas E, Porcheret M, Foster NE (2015). Maximising response from GPs to questionnaire surveys: do length or incentives make a difference?. BMC Med Res Methodol.

[39] Ziegenfuss JY, Niederhauser BD, Kallmes D, Beebe TJ (2013). An assessment of incentive versus survey length trade-offs in a Web survey of radiologists. J Med Internet Res.

[40] Van Otterloo J, Richards JL, Seib K, Weiss P, Omer SB (2011). Gift card incentives and non-response bias in a survey of vaccine providers: the role of geographic and demographic factors. PLoS One.

[41] Eysenbach Gunther, Consort- EHEALTHGroup (2011). CONSORT-EHEALTH: improving and standardizing evaluation reports of Web-based and mobile health interventions. J Med Internet Res.

