# Supplementary material for: Allowing Physicians to Choose the Value of Compensation for Participation in a Web-Based Survey: Randomized Controlled Trial
Source: J Med Internet Res. 2015 Jul 29;17(7):e189. doi: 10.2196/jmir.3898 (PMC4705363; doi:10.2196/jmir.3898)
Supplement: Multimedia Appendix 1 [file jmir_v17i7e189_app1.pdf]

CONSORT-EHEALTH Checklist V1.6.2 Report

(based on CONSORT-EHEALTH V1.6), available at [http://tinyurl.com/consort-ehealth-v1-6].

3898

Date completed

9/27/2014 22:20:47

by

Alison Turnbull

Allowing physicians to choose the value of compensation for participation in a web-based survey: a randomized controlled trial

TITLE

1a-i) Identify the mode of delivery in the title

"Allowing physicians to choose the value of compensation for participation in a web-based survey: a randomized controlled trial"

1a-ii) Non-web-based components or important co-interventions in title

There were no non-web-based components.

1a-iii) Primary condition or target group in the title

"Allowing physicians to choose the value of compensation for participation in a web-based survey: a randomized controlled trial"

ABSTRACT

1b-i) Key features/functionalities/components of the intervention and comparator in the METHODS section of the ABSTRACT

"The code could be instantly redeemed for an amount chosen by the respondent,"

1b-ii) Level of human involvement in the METHODS section of the ABSTRACT

There was no human involvement.

1b-iii) Open vs. closed, web-based (self-assessment) vs. face-to-face assessments in the METHODS section of the ABSTRACT

Participants completed "a short web-based survey"
and were recruited "from an existing database" via "a survey invitation e-mail."

1b-iv) RESULTS section in abstract must contain use data

"The overall response rate was 39%."

1b-v) CONCLUSIONS/DISCUSSION in abstract for negative trials

"Permitting intensive care physicians to determine compensation level for completing a short web-based survey modestly increased response rate and substantially decreased response time without decreasing the time spent on survey completion."

INTRODUCTION

2a-i) Problem and the type of system/solution

"[1]. However, surveys of healthcare providers are plagued by declining response rates [2–4], and techniques for increasing response rates in the general public [5] frequently fail to motivate physicians [6]."

"A major challenge when designing a survey is determining the appropriate level of financial compensation required to incentivize participation [16–18]."

2a-ii) Scientific background, rationale: What is known about the (type of) system

"Although physician response rates to electronic surveys have generally been lower than to postal surveys, many trials comparing postal versus electronic surveys were conducted a decade ago and targeted community-based physicians in regions were high-speed internet access was unreliable or nonexistent [12–15]."

METHODS

3a) CONSORT: Description of trial design (such as parallel, factorial) including allocation ratio

"We combined three techniques to address these challenges. First, we invited physicians to select their preferred level of instant compensation, up to $50, for completing a short, electronic survey. Second, we attempted to engender altruism by reminding physicians that the study was funded by a limited student budget. Finally, compensation was only promised to the first 100 respondents, making it a scarce, time-limited incentive. To assess whether these three combined techniques affect response rate, time to response, survey completion rate, and time spent completing the electronic survey, we designed a randomized controlled trial of respondent-selected compensation."

3b) CONSORT: Important changes to methods after trial commencement (such as eligibility criteria), with reasons

There were no changes to the methods after trial commencement.

3b-i) Bug fixes, Downtimes, Content Changes

There was no downtime and there were no unexpected events.

4a) CONSORT: Eligibility criteria for participants

"A previously described database of academic intensivists was used to recruit faculty from U.S. hospitals with training programs accredited by the Accreditation Council for Graduate Medical Education in Internal Medicine-Critical Care Medicine, Anesthesiology-Critical Care Medicine, and Surgical Critical Care [21]. "

"Physicians were excluded from randomization if they 1) lacked electronic contact information (11%), 2) had been invited to participate in a pilot study (11%), 3) had made a previous request not to be contacted (3%), or 4) contributed to study design or survey development (<1%)."

4a-i) Computer / Internet literacy

All participants were critical care physicians at teaching hospitals. This is a population whose profession requires computer/internet literacy.

4a-ii) Open vs. closed, web-based vs. face-to-face assessments:

"A previously described database of academic intensivists was used to recruit faculty from U.S. hospitals with training programs accredited by the Accreditation Council for Graduate Medical Education in Internal Medicine-Critical Care Medicine, Anesthesiology-Critical Care Medicine, and Surgical Critical Care [21]. The database was updated in 2012 to include demographic and electronic contact information for 2,482 physicians."

"On November 20, 2012, each randomized intensivist was sent an invitation by e-mail containing a unique link to the survey."

4a-iii) Information giving during recruitment

"The institutional review board of Johns Hopkins School of Medicine approved the study. Intensivists were notified that survey completion served as consent to participate in the trial."

"All invitations included the survey topic, number of questions, expected time required to complete survey, IRB approval, study confidentiality, number of follow-up/reminder e-mails for non-responders, planned date for study closing (December 20th, 2012), and names and affiliations of study investigators."

4b) CONSORT: Settings and locations where the data were collected

"A previously described database of academic intensivists was used to recruit faculty from U.S. hospitals with training programs accredited by the Accreditation Council for Graduate Medical Education in Internal Medicine-Critical Care Medicine, Anesthesiology-Critical Care Medicine, and Surgical Critical Care [21]."

4b-i) Report if outcomes were (self-)assessed through online questionnaires

"All surveys contained two questions assessing study eligibility followed by 10 screens each containing a brief clinical scenario for review."

4b-ii) Report how institutional affiliations are displayed

"All invitations included the survey topic, number of questions, expected time required to complete survey, IRB approval, study confidentiality, number of follow-up/reminder e-mails for non-responders, planned date for study closing (December 20th, 2012), and names and affiliations of study investigators."

5) CONSORT: Describe the interventions for each group with sufficient details to allow replication, including how and when they were actually administered

5-i) Mention names, credential, affiliations of the developers, sponsors, and owners

"Invitations for intensivists randomized to receive an incentive to participate also included the following text:
“In appreciation for your participation, the first 100 respondents to complete the survey will be offered an Amazon.com gift code at the end of the survey. The code can be redeemed immediately for any amount up to $50. In selecting the compensation amount, please consider that this is a PhD thesis project being funded by a limited student budget.”
Reminder e-mails were sent to all intensivists who had not completed the survey on days 13, 22, and 28 following the initial invitation. In each of the reminder e-mails, intensivists randomized to the incentive group were informed that funds for gift code creation were still available."

"Participants randomized to the incentive intervention who completed the survey had the option of entering the amount they wished to spend at Amazon.com® up to $50 USD using the Amazon Gift Codes On DemandTM service. Participants who entered a denomination were shown a claim code on the screen which was immediately redeemable on the Amazon.com® website."

5-ii) Describe the history/development process

"The database was updated in 2012 to include demographic and electronic contact information for 2,482 physicians."

"Physicians were excluded from randomization if they 1) lacked electronic contact information (11%), 2) had been invited to participate in a pilot study (11%), 3) had made a previous request not to be contacted (3%), or 4) contributed to study design or survey development (<1%)."

5-iii) Revisions and updating

There were no updates to the survey and no changing content.

5-iv) Quality assurance methods

Not applicable. The content of physician response was not a study outcome and only opinions were solicited.

5-v) Ensure replicability by publishing the source code, and/or providing screenshots/screen-capture video, and/or providing flowcharts of the algorithms used

"Invitations for intensivists randomized to receive an incentive to participate also included the following text:
“In appreciation for your participation, the first 100 respondents to complete the survey will be offered an Amazon.com gift code at the end of the survey. The code can be redeemed immediately for any amount up to $50. In selecting the compensation amount, please consider that this is a PhD thesis project being funded by a limited student budget.”"

5-vi) Digital preservation

Qualtrics Survey Software http://www.qualtrics.com Archived at: http://www.webcitation.org/6SuFdsRPO

5-vii) Access

"On November 20, 2012, each randomized intensivist was sent an invitation by e-mail containing a unique link to the survey. All invitations included the survey topic, number of questions, expected time required to complete survey, IRB approval, study confidentiality, number of follow-up/reminder e-mails for non-responders, planned date for study closing (December 20th, 2012), and names and affiliations of study investigators. Invitations for intensivists randomized to receive an incentive to participate also included the following text:
“In appreciation for your participation, the first 100 respondents to complete the survey will be offered an Amazon.com gift code at the end of the survey. The code can be redeemed immediately for any amount up to $50. In selecting the compensation amount, please consider that this is a PhD thesis project being funded by a limited student budget.”
Reminder e-mails were sent to all intensivists who had not completed the survey on days 13, 22, and 28 following the initial invitation. In each of the reminder e-mails, intensivists randomized to the incentive group were informed that funds for gift code creation were still available. Because not all respondents who created a gift code chose to take the full amount available, there were sufficient funds to offer the incentive to more than 100 respondents. All surveys contained two questions assessing study eligibility followed by 10 screens each containing a brief clinical scenario for review.
Participants randomized to the incentive intervention who completed the survey had the option of entering the amount they wished to spend at Amazon.com® up to $50 USD using the Amazon Gift Codes On DemandTM service. Participants who entered a denomination were shown a claim code on the screen which was immediately redeemable on the Amazon.com® website. The value of created gift codes was instantly deducted from a study fund containing $5,000. Study investigators could not access information on goods purchased by participants or the timing of purchases made using gift codes."

5-viii) Mode of delivery, features/functionalities/components of the intervention and comparator, and the theoretical framework

Not relevant to this study.

5-ix) Describe use parameters

"All invitations included the survey topic, number of questions, expected time required to complete survey (5 minutes), - "

5-x) Clarify the level of human involvement

Not relevant.

5-xi) Report any prompts/reminders used

"Reminder e-mails were sent to all intensivists who had not completed the survey on days 13, 22, and 28 following the initial invitation. In each of the reminder e-mails, intensivists randomized to the incentive group were informed that funds for gift code creation were still available."

5-xii) Describe any co-interventions (incl. training/support)

None.

6a) CONSORT: Completely defined pre-specified primary and secondary outcome measures, including how and when they were assessed

"Primary and secondary outcomes
The overall response rate among intensivists offered an incentive was 42% (359 / 859) versus 37% (367 / 991) in the control group (P = .04) (Figure 1). The proportion of respondents answering all survey questions was modestly greater in the incentive group (94% vs 90%, P = .06). In contrast to these relatively small effects on response, the incentive was associated with a large reduction in median time to response among responders (0.86 days and 6.4 days, P = .006; see Table 2). The median time required to complete the survey was 3.8 minutes in each group (P = .37)."

6a-i) Online questionnaires: describe if they were validated for online use and apply CHERRIES items to describe how the questionnaires were designed/deployed

Questionnaires were piloted in 2011 but responses to questionnaires were not the main survey outcome.

6a-ii) Describe whether and how “use” (including intensity of use/dosage) was defined/measured/monitored

"The primary outcome measure was the overall survey response rate, which was calculated as the number of intensivists who clicked the link in the invitation e-mail and viewed the first screen of the survey divided by the number of intensivists who were sent an invitation e-mail. Secondary outcome measures were defined as follows: survey completion rate (number of intensivists who answered all survey questions divided by the number of intensivists who clicked the link to the web-based survey); time to response (time of survey completion minus the time the initial email was sent among intensivists who completed the survey); and time spent completing survey (time of survey completion minus time that the link in the invitation e-mail was clicked)."

6a-iii) Describe whether, how, and when qualitative feedback from participants was obtained

No qualitative feedback was obtained.

6b) CONSORT: Any changes to trial outcomes after the trial commenced, with reasons

None.

7a) CONSORT: How sample size was determined

7a-i) Describe whether and how expected attrition was taken into account when calculating the sample size

All physicians (in the USA) who met inclusion criteria were eligible. No sample was taken.

7b) CONSORT: When applicable, explanation of any interim analyses and stopping guidelines

None.

8a) CONSORT: Method used to generate the random allocation sequence

Randomization was performed using the R programming language.

8b) CONSORT: Type of randomisation; details of any restriction (such as blocking and block size)

"Randomization was blocked on intensivist sex, specialty (medicine, anesthesiology, or surgery), years since completing residency, and geographic region of residency [24,25]. Within each block 45% of eligible intensivists were randomly assigned to the ability to select their preferred level of compensation as an incentive to participate."

9) CONSORT: Mechanism used to implement the random allocation sequence (such as sequentially numbered containers), describing any steps taken to conceal the sequence until interventions were assigned

"Invitations for intensivists randomized to receive an incentive to participate also included the following text:
“In appreciation for your participation, the first 100 respondents to complete the survey will be offered an Amazon.com gift code at the end of the survey. The code can be redeemed immediately for any amount up to $50. In selecting the compensation amount, please consider that this is a PhD thesis project being funded by a limited student budget.”"

10) CONSORT: Who generated the random allocation sequence, who enrolled participants, and who assigned participants to interventions

"Randomization was blocked on intensivist sex, specialty (medicine, anesthesiology, or surgery), years since completing residency, and geographic region of residency [24,25]. Within each block 45% of eligible intensivists were randomly assigned to the ability to select their preferred level of compensation as an incentive to participate.
On November 20, 2012, each randomized intensivist was sent an invitation by e-mail containing a unique link to the survey."

11a) CONSORT: Blinding - If done, who was blinded after assignment to interventions (for example, participants, care providers, those assessing outcomes) and how

11a-i) Specify who was blinded, and who wasn’t

Participants

11a-ii) Discuss e.g., whether participants knew which intervention was the “intervention of interest” and which one was the “comparator”

It's possible that physicians talked to colleagues at the same institution and discovered that some of them had been offered the opportunity to create a gift code.

11b) CONSORT: If relevant, description of the similarity of interventions

"All invitations included the survey topic, number of questions, expected time required to complete survey (5 minutes), IRB approval, study confidentiality, number of follow-up/reminder e-mails for non-responders, planned date for study closing (December 20th, 2012), and names and affiliations of study investigators. Invitations for intensivists randomized to receive an incentive to participate also included the following text:
“In appreciation for your participation, the first 100 respondents to complete the survey will be offered an Amazon.com gift code at the end of the survey. The code can be redeemed immediately for any amount up to $50. In selecting the compensation amount, please consider that this is a PhD thesis project being funded by a limited student budget.”"

12a) CONSORT: Statistical methods used to compare groups for primary and secondary outcomes

"Analyses were performed using the R programming language version 3.0.1 (Vienna, Austria) [26] using two-sided significance tests, with $P < 0.05$ indicating statistical significance, and analyzed on an intention-to-treat basis. Hypothesis tests for differences in proportion were performed using Pearson’s chi-squared test. Fisher’s exact test was used when a cell within a contingency table contained fewer than 10 observations. Differences in the distribution of continuous variables were assessed using the Wilcoxon-Mann-Whitney test [27]. "

12a-i) Imputation techniques to deal with attrition / missing values

"Secondary outcome measures were defined as follows: survey completion rate (number of intensivists who answered all survey questions divided by the number of intensivists who clicked the link to the web-based survey)"

"The proportion of respondents answering all survey questions was modestly greater in the incentive group (94% vs 90%, P = .06). In contrast to these relatively small effects on response, the incentive was associated with a large reduction in median time to response among responders (0.86 days and 6.4 days, P = .006; see Table 2). "

12b) CONSORT: Methods for additional analyses, such as subgroup analyses and adjusted analyses

"Out of 336 intensivists who answered all survey questions and were offered a gift code, 111 (33%) chose to create one (Table 3). All who chose to create a gift code were able to do so, as $95 remained at study end from the $5000 originally budgeted for incentives. Overall, 80% of gift codes were created for the maximum value of $50. Among the 22 intensivists who created gift codes for less than $50, the median value was $20, (IQR = $11 - $25). Intensivists randomized to the incentive who created, versus did not create, gift codes completed their first residency more recently (median years since residency 15 vs. 21, P = .001). A greater proportion of male versus female intensivists chose to create gift codes (36% versus 21%, P = .04)."

RESULTS

13a) CONSORT: For each group, the numbers of participants who were randomly assigned, received intended treatment, and were analysed for the primary outcome

See figure 1.

"The overall response rate was 39% (726 / 1,850), with 92% (665 / 726) of respondents answering all questions in the survey (Figure 1)."

13b) CONSORT: For each group, losses and exclusions after randomisation, together with reasons

Again see Figure 1 which is the consort flow diagram.

13b-i) Attrition diagram

"The overall response rate was 39% (726 / 1,850), with 92% (665 / 726) of respondents answering all questions in the survey (Figure 1). The median time to response was 2.9 days (IQR = 0.24 – 22) and the median amount of time spent completing the survey was 3.8 minutes (IQR = 2.4 – 5.5) among responding participants. "

14a) CONSORT: Dates defining the periods of recruitment and follow-up

"On November 20, 2012, each randomized intensivist was sent an invitation by e-mail containing a unique link to the survey. All invitations included the survey topic, number of questions, expected time required to complete survey (5 minutes), IRB approval, study confidentiality, number of follow-up/reminder e-mails for non-responders, planned date for study closing (December 20th, 2012), and names and affiliations of study investigators."

14a-i) Indicate if critical “secular events” fell into the study period

"The observed association between instant compensation and time to response is likely to have been influenced by the perceived scarcity of the compensation (i.e. invitation email said that incentive offered to the first 100 respondents), and the proximity of the study timing to annual holiday spending in December."

14b) CONSORT: Why the trial ended or was stopped (early)

"planned date for study closing (December 20th, 2012)"
The trial ended as planned.

15) CONSORT: A table showing baseline demographic and clinical characteristics for each group

Yes. See Table 1.

15-i) Report demographics associated with digital divide issues

"Intensivists randomized to the incentive who created, versus did not create, gift codes completed their first residency more recently (median years since residency 15 vs. 21, P = .001). A greater proportion of male versus female intensivists chose to create gift codes (36% versus 21%, P = .04)."

"Among intensivists offered the gift code, the only respondent characteristics associated with taking it was time since completing residency and gender. More recent graduates of medical training are likely to have lower salaries, higher educational debt levels and greater electronic expertise, making $50 more valuable and accessible. Although previous studies have found male healthcare workers less likely to respond to surveys than women [2,10], a sex-based difference in response to compensation has not been commonly reported in prior literature and merits greater investigation."

16a) CONSORT: For each group, number of participants (denominator) included in each analysis and whether the analysis was by original assigned groups

16-i) Report multiple “denominators” and provide definitions

"The primary outcome measure was the overall survey response rate, which was calculated as the number of intensivists who clicked the link in the invitation e-mail and viewed the first screen of the survey divided by the number of intensivists who were sent an invitation e-mail. Secondary outcome measures were defined as follows: survey completion rate (number of intensivists who answered all survey questions divided by the number of intensivists who clicked the link to the web-based survey); time to response (time of survey completion minus the time the initial email was sent among intensivists who completed the survey); and time spent completing survey (time of survey completion minus time that the link in the invitation e-mail was clicked)."

"Analyses were performed using the R programming language version 3.0.1 (Vienna, Austria) [26] using two-sided significance tests, with P < 0.05 indicating statistical significance, and analyzed on an intention-to-treat basis."

"The overall response rate was 39% (726 / 1,850), with 92% (665 / 726) of respondents answering all questions in the survey (Figure 1)."

16-ii) Primary analysis should be intent-to-treat

"Analyses were performed using the R programming language version 3.0.1 (Vienna, Austria) [26] using two-sided significance tests, with P < 0.05 indicating statistical significance, and analyzed on an intention-to-treat basis."

17a) CONSORT: For each primary and secondary outcome, results for each group, and the estimated effect size and its precision (such as 95% confidence interval)

No effect sizes were calculated.

17a-i) Presentation of process outcomes such as metrics of use and intensity of use

"In contrast to these relatively small effects on response, the incentive was associated with a large reduction in median time to response among responders (0.86 days and 6.4 days, P = .006; see Table 2). The median time required to complete the survey was 3.8 minutes in each group (P = .37)."

". Overall, 80% of gift codes were created for the maximum value of $50. Among the 22 intensivists who created gift codes for less than $50, the median value was $20, (IQR = $11 - $25). Intensivists randomized to the incentive who created, versus did not create, gift codes completed their first residency more recently (median years since residency 15 vs. 21, P = .001). A greater proportion of male versus female intensivists chose to create gift codes (36% versus 21%, P = .04)."

17b) CONSORT: For binary outcomes, presentation of both absolute and relative effect sizes is recommended

Absolute effect sizes are reported.

18) CONSORT: Results of any other analyses performed, including subgroup analyses and adjusted analyses, distinguishing pre-specified from exploratory

No subgroup or adjusted analyses were performed.

18-i) Subgroup analysis of comparing only users

If you count completing the survey as "using" then the following is a subgroup analysis of the users in the intervention group.

"Out of 336 intensivists who answered all survey questions and were offered a gift code, 111 (33%) chose to create one (Table 3). All who chose to create a gift code were able to do so, as $95 remained at study end from the $5000 originally budgeted for incentives. Overall, 80% of gift codes were created for the maximum value of $50. Among the 22 intensivists who created gift codes for less than $50, the median value was $20, (IQR = $11 - $25). Intensivists randomized to the incentive who created, versus did not create, gift codes completed their first residency more recently (median years since residency 15 vs. 21, P = .001). A greater proportion of male versus female intensivists chose to create gift codes (36% versus 21%, P = .04)."

19) CONSORT: All important harms or unintended effects in each group

None.

19-i) Include privacy breaches, technical problems

None.

19-ii) Include qualitative feedback from participants or observations from staff/researchers

None.

DISCUSSION

20) CONSORT: Trial limitations, addressing sources of potential bias, imprecision, multiplicity of analyses

20-i) Typical limitations in ehealth trials

"A potential limitation of our study is the generalizability of our results to other groups of physicians and other healthcare providers."

21) CONSORT: Generalisability (external validity, applicability) of the trial findings

21-i) Generalizability to other populations

"A potential limitation of our study is the generalizability of our results to other groups of physicians and other healthcare providers."

21-ii) Discuss if there were elements in the RCT that would be different in a routine application setting

None.

22) CONSORT: Interpretation consistent with results, balancing benefits and harms, and considering other relevant evidence
22-i) Restate study questions and summarize the answers suggested by the data, starting with primary outcomes and process outcomes (use)
"In a national randomized trial of 1,850 academic intensivists, permitting these physicians to choose their preferred level of financial compensation for participating in a short web-based survey resulted in a 5% absolute (13% relative) increase in response rate, a 4% absolute increase in survey completeness, and a faster response time (0.86 vs. 6.4 days), with no impact on the time spent completing the survey. Although 67% of intensivists offered compensation did not take it, those who did accept it generally took the maximum $50 amount that was available to them."

22-ii) Highlight unanswered new questions, suggest future research
"Although previous studies have found male healthcare workers less likely to respond to surveys than women [2,10], a sex-based difference in response to compensation has not been commonly reported in prior literature and merits greater investigation."

"Determining the appropriate amount of compensation to offer for survey completion remains challenging. Given that the vast majority of respondents who elected compensation took the maximum amount suggests that $50 may not have been viewed as sufficient by the majority of intensivists requiring a financial incentive to participate in this very short survey. Future studies with the ability to offer greater incentives and thus subject to less of a ceiling effect could provide insight into the distribution of preferred compensation for survey participation among physicians."

Other information

23) CONSORT: Registration number and name of trial registry
This trial did not enroll patients and does not have a name or number.

24) CONSORT: Where the full trial protocol can be accessed, if available
The full trial protocol is not available elsewhere.

25) CONSORT: Sources of funding and other support (such as supply of drugs), role of funders
"AET received support from the Johns Hopkins University Sommer Scholars Program and a postdoctoral training grant from the National Institute on Aging, T32AG000247. Financial support for this work was provided by a Doctoral Thesis Research Fund award from the Department of Epidemiology, Johns Hopkins University Bloomberg School of Public Health."

X26-i) Comment on ethics committee approval
"The institutional review board of Johns Hopkins School of Medicine approved the study."

x26-ii) Outline informed consent procedures
" Intensivists were notified that survey completion served as consent to participate in the trial. "

X26-iii) Safety and security procedures
The invitation e-mail contained the following privacy and security information:

"If you choose to participate, you will be assigned an identification (ID) number. This ID number, rather than your email address, will be linked to the survey data. We will not use your name or email address during data analysis or in any reports of the research findings. All information collected as part of this research study will only be presented in aggregate form. Information about individuals or their institutions will not be divulged. Your e-mail address was obtained from a database of intensive care physicians at academic teaching hospitals. Your participation is completely voluntary and is greatly appreciated."

X27-i) State the relation of the study team towards the system being evaluated
None.
